# Engineering tyrosine electron transfer pathways decreases oxidative toxicity in hemoglobin: implications for blood substitute design

**DOI:** 10.1042/BCJ20160243

**Published:** 2016-09-27

**Authors:** Gary G.A. Silkstone, Rebecca S. Silkstone, Michael T. Wilson, Michelle Simons, Leif Bülow, Kristian Kallberg, Khuanpiroon Ratanasopa, Luca Ronda, Andrea Mozzarelli, Brandon J. Reeder, Chris E. Cooper

**Affiliations:** 1 School of Biological Sciences, University of Essex, Wivenhoe Park, Colchester, Essex CO4 3SQ, U.K.; 2 Department of Pure and Applied Biochemistry, Lund University, Box 124, 221 00 Lund, Sweden; 3 Department of Neurosciences, University of Parma, Parma, Italy; 4 Department of Pharmacy, University of Parma, Parma, Italy; 5 Institute of Biophysics, National Research Council (CNR), Pisa, Italy

**Keywords:** hemoglobin, oxidative stress, reactive oxygen species

## Abstract

Hemoglobin (Hb)-based oxygen carriers (HBOC) have been engineered to replace or augment the oxygen-carrying capacity of erythrocytes. However, clinical results have generally been disappointing due to adverse side effects linked to intrinsic heme-mediated oxidative toxicity and nitric oxide (NO) scavenging. Redox-active tyrosine residues can facilitate electron transfer between endogenous antioxidants and oxidative ferryl heme species. A suitable residue is present in the α-subunit (Y42) of Hb, but absent from the homologous position in the β-subunit (F41). We therefore replaced this residue with a tyrosine (βF41Y, Hb Mequon). The βF41Y mutation had no effect on the intrinsic rate of lipid peroxidation as measured by conjugated diene and singlet oxygen formation following the addition of ferric(met) Hb to liposomes. However, βF41Y significantly decreased these rates in the presence of physiological levels of ascorbate. Additionally, heme damage in the β-subunit following the addition of the lipid peroxide hydroperoxyoctadecadieoic acid was five-fold slower in βF41Y. NO bioavailability was enhanced in βF41Y by a combination of a 20% decrease in NO dioxygenase activity and a doubling of the rate of nitrite reductase activity. The intrinsic rate of heme loss from methemoglobin was doubled in the β-subunit, but unchanged in the α-subunit. We conclude that the addition of a redox-active tyrosine mutation in Hb able to transfer electrons from plasma antioxidants decreases heme-mediated oxidative reactivity and enhances NO bioavailability. This class of mutations has the potential to decrease adverse side effects as one component of a HBOC product.

## Introduction

Hemoglobin (Hb)-based oxygen carriers (HBOC, colloquially termed ‘blood substitutes’) have the potential to be transfused in place of packed red blood cells to restore impaired oxygen transport [1]. HBOC offer the potential advantages of universal compatibility, enhanced shelf life, no risk of disease transmission, enhanced and controllable oxygen delivery, improved rheological properties, and more reliable availability. They are also available to individuals who cannot receive conventional blood transfusions for clinical or religious reasons. HBOC have also been used in addition to standard therapy in attempts to augment oxygen delivery in the microvasculature.

There have been a variety of different approaches to optimize recombinant Hb as a suitable starting material for a HBOC product [2]. Historically, Olson's group has been instrumental in introducing a variety of globin mutants to enhance stability, increase affinity, and decrease nitric oxide (NO) dioxygenase activity [2,3]. Nagai and co-workers [4] engineered genetically linked Hb dimers to increase vascular retention time and also created new interactions with plasma effectors, such as bicarbonate, to enhance oxygen offloading in tissue [5]. Many groups used novel cysteine residues to create sulfhydryl bridges and stabilize higher order protein structures [6,7].

In a recent review bemoaning the lack of viable approved products, blame was largely placed on side effects due to the redox reactivity of Hb [1]. However, to date, specific issues relating to the oxidative stress induced by recombinant Hb have focused almost entirely on two different areas. First, avoiding significant increases in the autoxidation rate — the conversion of oxyhemoglobin (oxyHb) to methemoglobin (metHb) and superoxide radical. Secondly, avoiding oxidation during the manufacturing/protein production process; for example, Levine et al. [8] showed that modifying the His-2 residue could decrease oxidative modification in the β-subunit and suggested that recombinant proteins that maintained their N-terminal methionine could have additional antioxidative properties [9].

There is a general acceptance [10] that cell-free Hb induces oxidative stress *in vivo* by reacting with peroxides (H_2_O_2_ or lipid-derived). In the ferrous state (oxy or deoxy), this creates ferryl iron; in the ferric (met) state, both ferryl iron and a globin radical are produced. Note, given that ferryl Hb is autoreduced back to ferric, the eventual production of globin radicals is inevitable even if the starting material contains negligible metHb. We have shown that the plasma antioxidants, ascorbate and urate, act synergistically to maintain extracellular Hb in a functional state [11].

Following peroxide addition to ferric globins, ascorbate can quantitatively capture both the ferryl iron and the free radical species [12]. However, kinetic limitations, or a decrease in antioxidant levels, may result in partial reduction and a consequent increase in tissue oxidative damage. Tyrosine residues are able to act as redox mediators by one-electron cycling between oxidized (radical) and reduced forms. Indeed, many enzymes, such as catalases and peroxidases, make use of tyrosine redox centers [13,14]. We have shown that myoglobin [15] and the Hb α-subunit [16] have electron transfer pathways that are able to enhance the rate of ferryl reduction by plasma antioxidants. These proteins have a high-affinity saturable pathway where the initial electron acceptor is a tyrosine residue. The Hb β-subunit lacks such a pathway [16]. Introducing such a pathway into the β-subunit in the homologous site where one is present in the α-subunit (βF41Y) resulted in enhanced ferryl reduction in tetrameric Hb [16].

Myoglobin from the sea hare (*Aplysia fasciata*) lacks any tyrosine residues. Therefore, like the β-subunit of human Hb, it lacks a high-affinity ferryl reduction pathway. In this model system, we were able to create a variety of phenylalanine to tyrosine mutations with enhanced electron transfer pathways for ascorbate [17]. Furthermore, in the presence of ascorbate, but not in its absence, these mutants had significantly decreased lipid peroxidation activity compared with the wild-type (wt) protein. The purpose of the present study was therefore to determine if introducing a new tyrosine-based electron transfer pathway in human Hb resulted in similar antioxidant properties.

**Figure 1.**
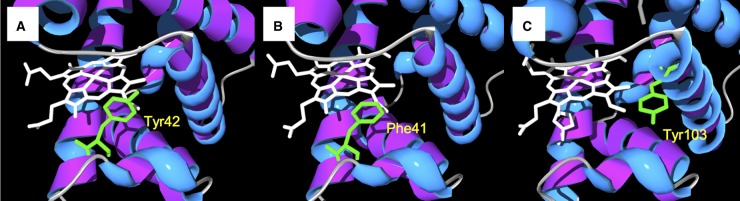
Tyrosine electron transfer pathways in globins. Active site structure of Hb α-subunit (**A**), Hb β-subunit (**B**), and myoglobin (**C**); sites of redox-active tyrosine residues are indicated. Note that in the Hb β-subunit, the tyrosine is substituted with a phenylalanine. Mutating this to a tyrosine (βF41Y) creates a new ascorbate reducible site. This diagram was adapted from a figure originally published in The Journal of Biological Chemistry. Reeder, B. J. et al. Tyrosine residues as redox cofactors in human hemoglobin: Implications for engineering non toxic blood substitutes. *J. Biol. Chem.* 2008; 283:30780–30787 © the American Society for Biochemistry and Molecular Biology.

We therefore determined the oxidative reactivity of the set of Hb mutants; we previously showed to differ in their tyrosine electron transfer pathways [16] (Figure 1). This involved creating a new electron transfer pathway in the β-subunit (βF41Y), deleting the existing one in the α-subunit (αY42V), and transferring the pathway from the α- to the β-chain (αY42V/βF41Y). The properties of recombinant βF41Y are particularly interesting as this mutation is present in the human polymorphism Hb Mequon [18].

## Experimental

### Protein preparation

The cloning, expression, and purification of the recombinant Hb proteins were carried out as previously described [16], and the proteins were stored in the stable ferrous-CO adduct forms. Native Hb was purified from adult volunteers [11]. To make ferric Hb, the CO forms were incubated with potassium ferricyanide (10 mM) at 4°C, and the CO was removed by constant strong light. The excess ferri/ferrocyanide was removed from the ferric proteins by filtration through a size-exclusion Sephadex G-25 column (∼5 × 0.5 cm). The concentration of Hb was determined by reduction in an aliquot of the ferric protein to the deoxy form using sodium dithionite and using the extinction coefficient of 133 mM^−1^ cm^−1^ at 430 nm [19]. The oxy form of the Hb proteins was made by the addition of a slight excess of sodium dithionite to the ferric proteins, followed by passage down a Sephadex G-25 column.

### HPLC analysis

Hb samples were analyzed by reverse-phase HPLC using an Agilent 1290 HPLC fitted with a diode array spectrophotometer. The column used was a Zorbax StableBond 300C3 250 mm × 4.6 mm fitted with a 12 mm × 4.6 mm guard column. Solvents were (A) 0.1% TFA (trifluoroacetic acid) and (B) acetonitrile-containing 0.1% TFA. The gradient was initially 35% solvent B, stable for 10 min, increasing to 37% solvent B over 5 min. This was increased to 40% solvent B over 1 min and then to 43% solvent B over 10 min. The flow rate was 1 ml/min, and the temperature was 25°C.

### Oxygen affinity measurements

OxyHb solutions were left overnight under helium flow and in the presence of the Hayashi reducing system before titrations [20]. The oxygen p50 and Hill coefficients were obtained by measuring optical spectra after equilibration at varying *p*O_2_ gas mixtures as previously described [21]. For *in vitro* conditions, p50 values were measured in 100 mM HEPES, 1 mM EDTA, pH 7.0, 15°C; conditions more akin to that in the vasculature (pseudo-physiological conditions) were 100 mM HEPES, 1 mM EDTA, 100 mM sodium chloride and 1.2 mM sodium phosphate, pH 7.0, 25°C. The fractional saturation was calculated by fitting the data to a linear combination of oxy, deoxy, and met Hb reference spectra.

### Liposome preparation

α-Phosphatidylcholine from soybean (Type II-S, from Sigma) was added to the reaction buffer (sodium phosphate, 20 mM, pH 7.4) at a concentration of 5 mg/ml and then sonicated in a water bath for 15 min, until no particulates could be seen. The final liposome solution was maintained a pearlescent appearance. This suspension was then forced through a Northen Lipids extruder containing a membrane of size cutoff 0.1 μm using 25 mm Whatman filtration membranes [22]. A minimum of 10 extrusions per sample preparation was used to produce unilamellar liposomes of ∼100 nm.

### Conjugated diene and singlet oxygen formation in liposomes

The concentration of ferric proteins used in all liposome oxidation experiments was 2 µM heme. The amount of extruded liposomes used in all lipid oxidation assays was ∼30 µl/ml. Lipid oxidation was measured in two ways using UV and fluorescence spectroscopies. Conjugated diene formation was measured by following the increase in absorbance at 234 nm using a Varian Cary 5E spectrophotometer. Data were collected every 7–10 min for up to 12 h. Sample volumes were 120–150 µl. Studies were performed in the presence and absence of physiological levels of ascorbate (30 µM). Dunnett's many-to-one comparison test was used to determine if a mutant was different from control. In addition, due to the variability in the lipid oxidation properties between liposome preparations, in separate experiments, each protein was also compared directly with each other in the same batch of liposomes (*n* = 3) using unpaired *t*-tests. Singlet oxygen (^1^O_2_) production was measured as fluorescence intensity of the fluorophore Singlet Oxygen Sensor Green (SOSG, Molecular Probes) using a FLUOstar OPTIMA fluorescence plate reader from BMG LABTECH. The stock solution of SOSG (5 mM in methanol) was diluted into the reaction mixture to a final concentration of 0.25 µM. The reactions containing SOSG were excited at 480 nm and emission was followed at 520 nm. Data were collected every ∼3 min for up to 12 h. Sample volumes were 200 µl.

### Autoxidation measurements

The rate of conversion of ferrous oxy Hb proteins to ferric Hb was monitored by UV–visible spectroscopy in 20 mM sodium phosphate (pH 7.4); for autoxidation measurements at 25°C, spectra (375–700 nm) were collected for up to 48 h. For autoxidation measurements at 37°C, whole spectra were collected for up to 3 h. Sample volumes were 1 ml. Protein concentrations were 10 µM heme. The kinetic traces were analyzed by fitting to single exponential fits.

### Reactions with the lipid hydroperoxide HPODE

The met forms of the Hb proteins were reacted with the 13-S hydroperoxy 9-*cis*, 11-*trans* octadecadienoic acid (HPODE) in a stopped-flow diode array spectrophotometer (Applied Photophysics, model SX-20). On rapid mixing of the proteins with HPODE, spectral changes in the Soret and visible regions were monitored. Heme destruction was monitored by the bleaching of the Soret peak at 405 nm. Time courses were analyzed by fitting to a double exponential curve, although in the presence of the β41 mutation the two rates were indistinguishable.

### NO dioxygenation rate

An NO solution was prepared by dissolving the NO donor Proli NONOate (Alexis Biochemicals) in a lightly buffered degassed solution at alkaline pH. The concentration of Proli NONOate was determined using an extinction coefficient of 8500 M^−1^ cm^−1^ at 250 nm [23]. Proli NONOate was then added to a strong buffer at neutral pH (100 mM sodium phosphate, pH 7.4) to release free NO gas; under these conditions, 1.8 molecules of NO are released per Proli NONOate molecule. The oxy form of the relevant Hb solution (in 100 mM sodium phosphate, pH 7.4) was placed in one syringe and the NO released from the Proli NONOate in the other. Both the solutions were then mixed rapidly in equal volumes in the stopped-flow spectrophotometer. After mixing, the Hb concentration was 4 μM and the NO concentration was 30 μM. The absorbance changes were monitored at single wavelengths of 405 and 430 nm over a 0.1 s time range. Owing to the speed of the reaction, the temperature was set to 15°C.

### Heme release from Hb

The vector pEMBL19-containing sperm whale myoglobin (SW Mb) H64Y/V67F was a kind gift from John Olsen (Rice University) [24] and was then His-tagged to simplify purification. The gene for SW Mb (H64Y/V67F) was amplified by overhanging PCR with the primers 5′-CCA**CATATG**GTTCTGTCTGAAGGTGAATGGCAGCTG and 5′-CAT**GGATCC**TCATTAACCCTGGTAACCCAGTTC (Eurofins) to introduce novel restriction sites for NdeI and BamHI (shown in bold). The PCR fragment was restricted with NdeI and BamHI then ligated into pET28a vector cut with the same restriction endonucleases to generate pET28a-SWMb (H64Y/V67F). To prepare His-tagged SW Mb (H64Y/V67F), BL21 (DE3) cells were first freshly transformed with the expression vector pET28a-SWMb (H64Y/V67F) and subsequently grown in Luria–Bertani media at 37°C to an optical density of ∼0.8 at 600 nm. Protein expression was then induced with 0.5 mM isopropyl β-d-1-thiogalactopyranoside, 0.25 mM aminolevulinic acid, and 0.1 mM ferric citrate. Cultures were then bubbled with pure CO gas, flasks sealed thoroughly with rubber bungs and grown for a further 18 h at 27°C and 90 rpm. Cells were then harvested by centrifugation at 4000 rpm for 20 min at 4°C. The cell paste was resuspended in 40 ml of buffer A [50 mM NaPi (pH 7.2) and 100 mM NaCl] and cells were lysed by using an Avestin C3 Emulsiflex homogenizer. The cell lysate was cleared by centrifugation at 18 000 rpm for 30 min at 4°C and the supernatant was filtered using 0.45 μm filters before being loaded onto a 5 ml HisTrap-HP column (GE Healthcare) pre-equilibrated with buffer A. The protein was eluted with a 50 ml linear gradient of buffer B [50 mM NaPi (pH 7.2), 100 mM NaCl, and 1 M imidazole] and fractions containing the myoglobin were pooled. The heme was then extracted to create apomyoglobin as described previously [25]. Heme release from the met forms of wt and βF41Y was then measured by incubating the proteins with an excess of the heme-binding apomyoglobin mutant H64Y/V67F and monitoring absorbance changes at 37°C in 100 mM NaPi pH 7.2, containing 0.15 M sucrose [24]. The fast phase of heme loss (β-subunit) was measured using single exponential fits to the first 60 s of data in a stopped-flow spectrophotometer looking at the appearance of the 600 nm peak. The slow phase (α-subunit) was also monitored at 600 nm using a Cary 5000 spectrophotometer, fitting to a double exponential and reporting the slower rate.

### Kinetic reactions of deoxy Hb with nitrite

Formation of the nitrosyl complexes from the deoxy forms of the recombinant Hb proteins wt and βF41Y was carried out in excess sodium dithionite [26]. Spectral changes were monitored in the Soret and visible regions, and time courses were fitted to 432–413 nm as single exponential fits (min^−1^) over a 20 min time range.

### Statistical analysis

The program KaleidaGraph 4 (Synergy Software) was used for analysis. In experiments involving two conditions, unpaired *t*-tests were used to determine significance. Where multiple tests or treatments were involved, ANOVA followed by Dunnett's many-to-one comparison test was used to determine if any condition was different from the control.

## Results

### Functional properties of the βF41Y mutant

**Figure 2.**
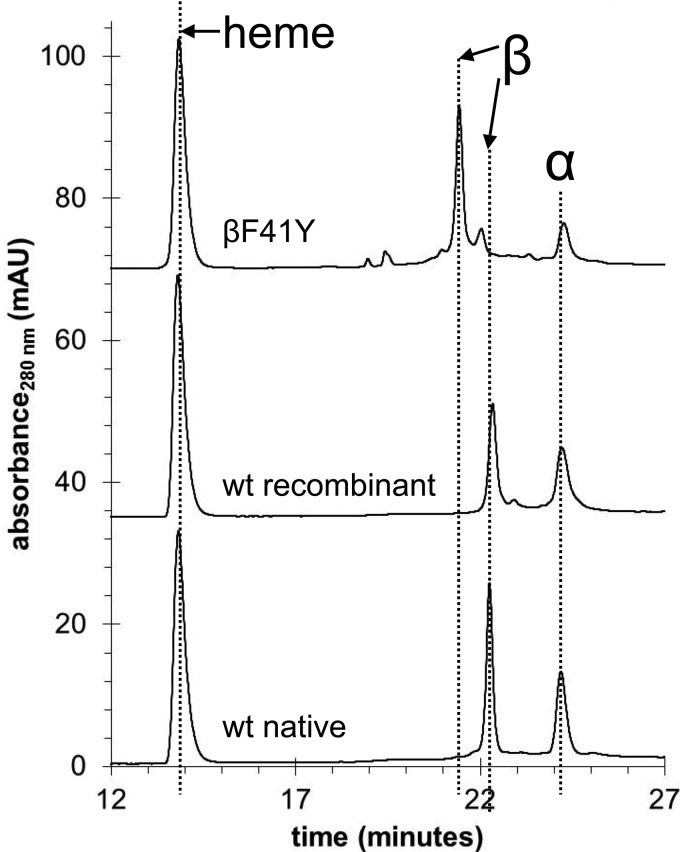
HPLC comparison of recombinant and native Hb. Reversed-phase HPLC separation of heme cofactor and protein subunits from native Hb, wt recombinant Hb and the βF41Y mutant.

Figure 2 shows reversed-phase HPLC data for the two recombinant proteins (wt and βF41Y) and native Hb purified from adult human volunteers. Although there is detectable heterogeneity in the recombinant proteins, the position of the α-peaks is identical in all three proteins and there is a distinct β-peak in βF41Y, consistent with the introduction of the mutation. All three Hb proteins showed very similar optical spectra in the deoxy, oxy, ferric, ferryl, and ferrous-CO forms.

In human Hb, amino acid residues 41 (β) and 42 (α) are located on the C7 helix close to the heme, but yet accessible to the external medium and the α-/β-subunit interface. Modifying residues close to the heme group or near the subunit interface might be expected to have an adverse effect on oxygen-binding characteristics of the protein [2]. However (Table 1), βF41Y exhibited an oxygen-binding affinity within 30% of wt, either in the absence of effector ligands or under ‘pseudo-physiological’ extracellular conditions (+1.2 mM phosphate and 100 mM Cl^−^).

**Table 1:** Functional properties of βF41Y Hb Mean ± SD (*n* = 3).

	Wt	βF41Y
Oxygen binding (intrinsic, *T* = 25°C)		
p50 (Torr)	2.61 ± 0.05	2.19 ± 0.04*
Hill coefficient	2.36 ± 0.06	1.36 ± 0.04*
Oxygen binding (+100 mM Cl^−^, *T* = 25°C)		
p50 (Torr)	5.21 ± 0.07	8.89 ± 0.51*
Hill coefficient	1.31 ± 0.02	1.37 ± 0.11*
Autoxidation (h^−1^), *T* = 25°C	0.13 ± 0.02	0.12 ± 0.01
Autoxidation (h^−1^), *T* = 37°C	0.44 ± 0.08	0.39 ± 0.09
NO dioxygenation (μM^−1^ s^−1^), *T* = 15°C	7.8 ± 0.2	6.4 ± 0.1*
MetHb heme off (min^−1^), *T* = 37°C (β-subunit)	1.04 ± 0.04	2.08 ± 0.25*
MetHb heme off (min^−1^), *T* = 37°C (α-subunit)	0.0055 ± 0.0016	0.0054 ± 0.0006

*P < 0.05 compared with wt. For detailed conditions, see the Experimental section.

Ferrous oxyHb is susceptible to autoxidation, producing the nonfunctional ferric (metHb) form and the superoxide radical. Enhanced autoxidation rates and accompanying heme loss from the ferric protein have the potential to prove problematic for HBOC applications [27]. The introduction of the βF41Y had no deleterious effect on autoxidation rate (Table 1), either at ambient temperature or at 37°C.

**Figure 3.**
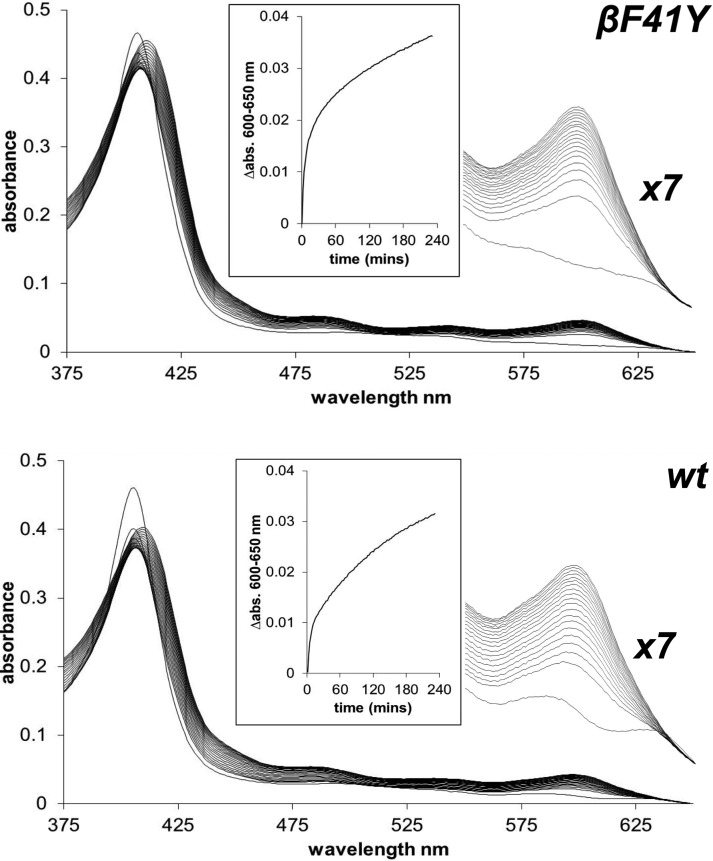
Heme loss from recombinant Hb. Heme loss from metHb and binding to the apo H64Y/V68F mutant. The spectral changes show the reaction following mixing metHb with the apo H64Y/V68F mutant for wt (bottom) and βF41Y (top). The main figure shows the spectral changes every 13 min from *t* = 0 to *t* = 4 h. An expanded region (×7) of the visible spectra in the 600 nm region is also shown. The inset shows the time course for the reaction in the visible region used for further kinetic analysis. Conditions: 100 mM NaPi, pH 7.2, containing 0.15 M sucrose; *T* = 37°C; [Hb] = 2.5 μM; [apo mbH64Y/V68F] = 30 μM.

However, the introduction of the βF41Y mutation did enhance heme release from metHb as measured by binding to the heme-binding apomyoglobin mutant H64Y/V67F (Figure 3). Optical spectra show a clear change in environment as the heme leaves Hb and binds to the mutant (as evidenced by the shift in the Soret peak and the loss of the 630 nm band). At the same time, there is a growth of absorbance in the visible region at 600 nm. Analysis of the rate of the rise in the peak revealed biphasic kinetics previously attributed to a fast loss of heme from the β-subunit and a lower loss from the α-subunit. Consistent with this assignment, only the fast phase showed increased heme loss in βF41Y (Table 1). The magnitude of this increase was an approximate doubling in rate.

**Figure 4.**
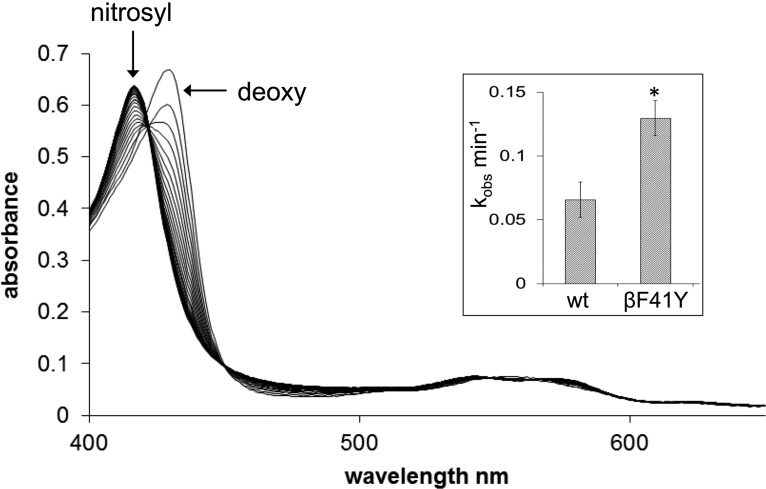
Hb nitrite reductase activity. Formation of nitrosyl Hb from deoxy Hb, wt, and βF41Y recombinant forms, in the presence of 0.2 mM nitrite. Conditions: sodium phosphate buffer (20 mM, pH 7.4, 25°C); [Hb] = 5 μM. Inset: the rate (mean ± SD, *n* = 6) of conversion from deoxy to nitrosyl. Asterisk indicates significant difference from wt (*P* < 0.0001).

A decrease in the *in vivo* levels of the vasodilator NO is a concern following HBOC administration due to scavenging by oxyHb. Mutations can be introduced into the heme pocket to decrease this rate by an order of magnitude [28]. Despite lacking these modifications, the NO dioxygenation rate was still slightly decreased in βF41Y (Table 1). More significantly, however, this was accompanied by a doubling in the rate of reduction in nitrite to NO catalyzed by deoxyHb (Figure 4).

### Lipid oxidation and oxidative stress

**Figure 5.**
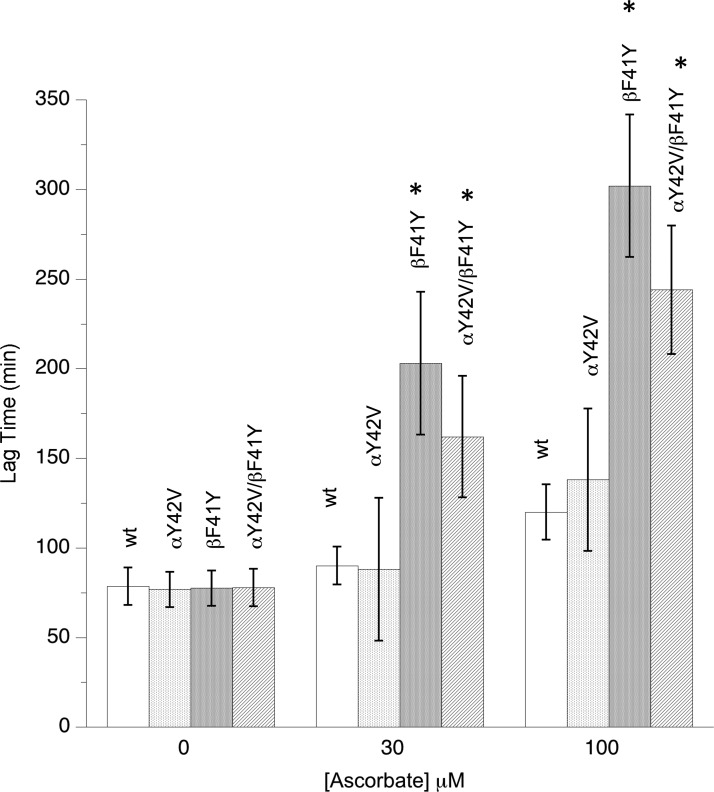
Effect of tyrosine mutations on Hb-catalyzed lipid peroxidation. Liposome oxidation monitored by conjugate diene formation following the addition of recombinant metHb in the presence or absence of ascorbate. Assay conditions: sodium phosphate (20 mM, pH 7.4, 30°C); [heme] = 2 µM; data presented as mean ± SD, *n* = 9. Asterisks indicate significant difference from wt (*P* < 0.05).

The addition of metHb to liposomes initiates lipid peroxidation by an autocatalytic reaction, the extent of the lag phase is a measure of the rate of lipid oxidation [17]. All the mutants oxidized synthetic liposomes at the same rate in the absence of ascorbate (Figure 5). However, in the presence of physiological levels of ascorbate, the addition of a tyrosine residue in the β-subunit significantly decreased oxidation rates. Additional competition studies directly comparing pairs of mutants revealed that the order of the onset of this lipid oxidation cascade in the presence of ascorbate was wt = αY42V < αY42V/βF41Y < βF41Y.

**Figure 6.**
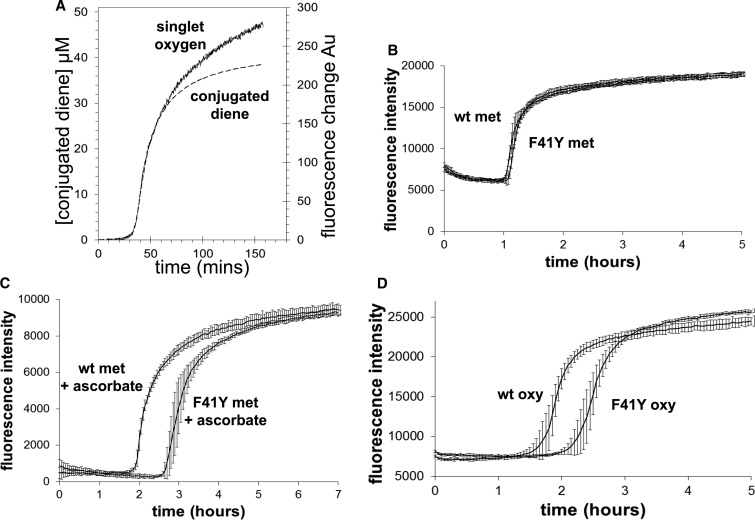
Singlet oxygen production catalyzed by recombinant Hb. Changes in singlet oxygen (fluorescence) and conjugated diene (absorbance) formation following the addition of metHb to liposomes (**A**). Comparison of wt and βF41Y metHb addition in the absence (**B**) and presence (**C**) of 50 μM ascorbate. Addition of wt and βF41Y oxyHb to liposomes (**D**). Assay conditions: sodium phosphate (20 mM, pH 7.4, 30°C); [heme] = 2 µM; [SOSG] = 0.25 µM. Data presented as mean ± SD, *n* = 8.

Lipid oxidation can also be measured by monitoring singlet oxygen production using the fluorescent dye SOSG (Figure 6A). The time course of lipid peroxidation following Hb addition to liposomes is identical whether following conjugated diene formation or singlet oxygen production. The rate of singlet oxygen production is identical in wt and βF41Y metHb (Figure 6B), but is decreased in the mutant in the presence of 50 μM ascorbate (Figure 6C). Following the addition of oxyHb, βF41Y is protective even in the absence of ascorbate (Figure 6D).

**Figure 7.**
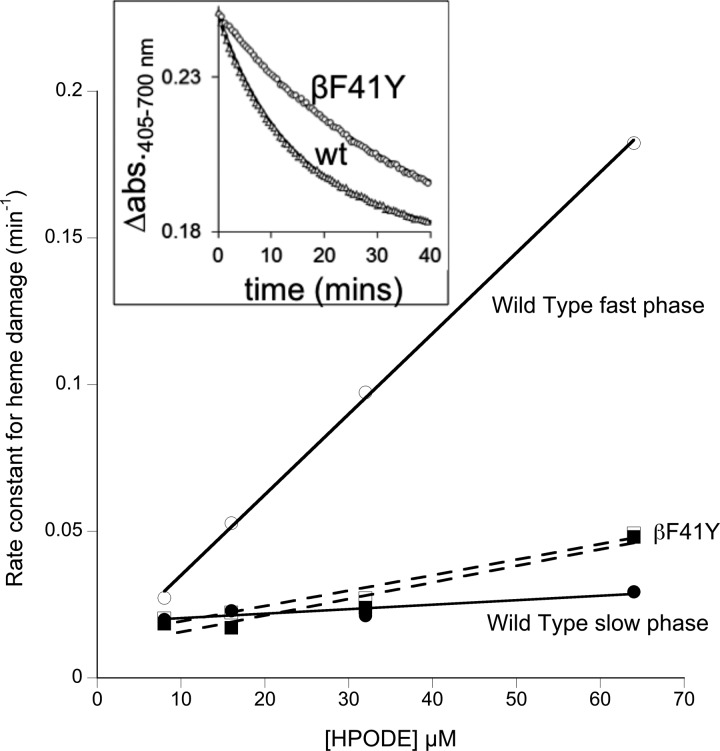
Reaction of the lipid peroxide HPODE with recombinant Hb. Addition of 32 μM HPODE to metHb (wt and βF41Y). Time courses of heme damage (inset) were fit to double exponentials and plotted against [HPODE]. Conditions: sodium phosphate (20 mM, pH 7.4); [heme] = 2 μM after mixing; *T* = 25°C.

Ferric globins react directly with the HPODE; a complex set of reactions follow, involving ferryl heme that ultimately leads to heme degradation [29]. Unlike in myoglobin, the kinetics of HPODE-induced heme degradation in Hb is biphasic, most likely due to preferential reactivity at the α- and β-subunits. The βF41Y mutant showed increased resistance to this heme degradation (Figure 7). Analysis of the HPODE concentration dependence revealed that the βF41Y effect was caused by removing the fast phase of damage, resulting in monoexponential rates of heme decay.

## Discussion

### Tyrosine mutations and lipid oxidation

The present study demonstrates the potential advantages of engineering tyrosine-based electron transfer pathways as a component of a HBOC, especially if combined with antioxidants. The exemplar protein studied was the βF41Y mutation, creating a novel protein with similar electron transfer sites facilitating ferryl reduction in both the α- and β-subunits. It is not *a priori* true that introducing a new pathway for electrons to enter and leave ferryl Hb will have new antioxidative properties; it could be argued that a new pathway could facilitate oxidative damage induced by Hb, making it easier for nearby lipids and proteins to be modified by the ferryl species. However, at least in the case of lipids, that did not seem to be the case. The onset of liposome oxidation by metHb was unchanged whether the electron transfer pathway was removed in the α-subunit or added to the β-subunit. However, in the presence of plasma antioxidants, such as ascorbate, the βF41Y mutation protected the liposomes from damage. This is consistent with our studies in *Aplysia* myoglobin, where we introduced two novel, fast, tyrosine-based electron transfer pathways [17]. Both of these protected against liposome oxidation, but again only in the presence of ascorbate.

Rather than being catalyzed by Hb ferric/ferryl redox cycling [10], liposome oxidation could be initiated directly by the ferric ion alone in metHb or indirectly via heme iron releasing from the protein and entering the lipid bilayer. The results in the present paper argue against this. Ascorbate is protective against lipid peroxidation in the presence of our mutants. Yet, ascorbate reduction in metHb [11] is slow compared with ascorbate reduction in ferryl Hb, and βF41Y does not enhance this already very slow rate (results not shown). In the case of heme insertion into the liposomes, the βF41Y shows slightly enhanced heme release (Table 1) and so, if anything, would be expected to be more pro-oxidative in this system.

There are two alternative mechanisms by which tyrosine mutations could increase the lag phase of lipid oxidation in the presence of ascorbate. The first is by direct reduction in the ferryl species that react with lipid peroxides. However, it is also theoretically possible that the tyrosine residue could provide a pathway whereby the unpaired electron on a lipid peroxide radical is transferred to ascorbate via Hb. In the latter case, electron transfer to/from the heme iron would not be directly involved. These mechanisms are not mutually incompatible and could operate synergistically. This could explain why in *Aplysia* Mb introducing new tyrosine residues enhances the reduction in ferryl Mb and decreases lipid oxidation, but the two activities do not closely correlate, that is, the mutant that reduces ferryl fastest is not the one that has the greatest inhibition of lipid oxidation. It could also explain why moving the tyrosine residue from α42 to β41 is partially protective in the presence of ascorbate (the present paper), despite only limited effects on the ferryl reduction rate [17].

Liposome oxidation catalyzed by oxyHb appears to be slower in βF41Y even in the absence of ascorbate. The reason for this could also be linked to the idea that βF41Y is in a particularly apposite environment to donate electrons. The reaction of oxyHb with liposomes is more complex than that of metHb, as not only does the heme react with lipid peroxides, but there is also a contribution from the autoxidation rate (which itself forms superoxide radicals). The autoxidation rate is slightly slower in βF41Y (Table 1) and this could at least partially explain the observed protection in the oxyHb mutant. However, there also seems to be a direct effect of lower reactivity with lipids. This is shown by the slower reaction of βF41Y metHb with the lipid peroxide HPODE. Globin reactions with HPODE are mechanistically complex [29]. Therefore, in the present paper, we limited ourselves to looking at the simpler endpoint of heme destruction. In wt Hb, this is biphasic. However, in the mutant, the faster phase is lost. The simplest explanation for this is that the presence of a redox-active tyrosine close to the heme enables the safe removal of an oxidizing species. Although we have previously suggested that the final stable protein radical following peroxide interaction with Hb is at βTyr145 [30], it is likely that the tyrosine 42 in the α-Hb subunit (or its homologous location in other globins) is the initial destination for the radical once it leaves the porphyrin ring [14,31,32]. Lacking such a tyrosine, the β-subunit may be more susceptible to heme destruction by HPODE. In this simple model, the biphasic reactivity seen in the present paper would be a simple consequence of differing reactivity at the α- and β-subunits, with the βF41Y mutation equalizing the relative rates.

Taken together, these results strongly suggest that the addition of a redox-active heme-accessible tyrosine to the β-subunit of human Hb would be a useful component of a future recombinant HBOC designed to decrease ferryl heme reactivity, especially when combined with a suitable reductant.

### βF41Y as a component of a HBOC

The βF41Y mutation is functional *in vivo* as it has been described in a human polymorphism (Hb Mequon). The clinical subject was not anemic, and the blood oxygen affinity was normal and sensitive to the effector BPG [18]. The Hb Mequon patient was suffering from severe hemolysis following a viral illness treated with acetaminophen. However, this was an isolated incident and none of the family relatives carried any hematological disorder. Although it is possible that there is some latent instability in Hb Mequon, it remains unclear whether there is any causal link between βF41Y and the hemolytic event. In any event, the existence of such a stable polymorphism in an otherwise healthy 34-year-old patient suggests that βF41Y is a relatively benign polymorphism. Although there may be subtle differences between the recombinant and native version of this protein, the data in the present paper support the idea that the βF41Y mutation is relatively benign; perhaps, the tendency for faster heme loss is ameliorated by enhanced antioxidative properties? In any event, there appears no reason *a priori* to dismiss βF41Y as a component of an acutely administered HBOC.

The oxygen affinity and autoxidation rate of βF41Y have been studied extensively under a range of conditions by the groups of Baudin-Creuza and Marden [33,34]. They showed a decrease in oxygen affinity compared with Hb A (the native protein purified from blood) and attributed this to stabilization of the T-state conformation. Our study is consistent with theirs in that a decrease in oxygen affinity was observed in the presence of 100 mM chloride. However, a slight increase in affinity in the absence of chloride was seen. Part of the difference is possibly because we are comparing our protein to the recombinant wt rather than Hb A; indeed, most of the difference seen in the absence of chloride is due to differences in our wt compared with their Hb A, rather than differences in the mutant itself. In general, when analyzing mutant Hbs, we caution against comparing solely against native Hb A, as recombinant proteins can be modified during growth or incompletely processed (for example, due to incomplete cleavage of the N-terminal methionine). In this regard, we found some reduction in co-operativity in βF41Y compared with previous publications [34]. It is possible that this was due to protein heterogeneity as in our hand values of the Hill coefficient in different preparations are more variable than those for the oxygen affinity; measurements on wt Hb from five different preparations showed robust p50 values under our ‘pseudo-physiological’ conditions (5.66 ± 0.42 mm Hg), but rather variable numbers for the Hill coefficient (2.23 ± 0.42).

In contrast with previous results, which showed a small increase in the autoxidation rate when compared with Hb A [35], we found that when compared with wt, βF41Y had a slightly decreased rate of autoxidation. Measurement of this rate is highly dependent on protein concentration and the nature of the analysis [35], and we would not put too much weight on these small differences. Of more concern for an HBOC is the rate of heme loss, which has not previously been measured in βF41Y. Heme loss from extracellular Hb is a concern in HBOC, particularly as free heme has recently been shown to act as a damage-associated pattern molecule, activating the immune response system via the TLR-4 receptor [36]. A small increase in this rate in the β-subunit was observed, which is of some concern. It is likely that this increased heme loss will need to be addressed in any final HBOC product, either by the additional mutants designed to ‘waterproof’ the heme pocket [37] or by subsequent chemical modifications. Alternatively, F41Y is not the only place, where a tyrosine can be introduced into the β-chain to enhance ferryl reduction. We have shown the same effect with another mutation introduced on the surface of Hb, βK66Y [38]; although this mutant is not oxidatively stable, it has decreased heme loss compared with wt. Therefore, the ability to enhance ferryl reduction does not automatically correlate with enhanced heme loss, suggesting that a different tyrosine mutation to βF41Y could be an even better starting material for a new HBOC.

It appears unlikely that surface-accessible tyrosine, even those close to the heme like βF41Y, would significantly affect the NO dioxygenation activity as this generally requires modification of the heme pocket [28]. This is indeed what was found; F41Y only showed a small decrease in NO scavenging. However, it did significantly enhance the nitrite reductase rate. Although this is known to be under allosteric control, and thus a function of the oxygen p50 [39,40], the βF41Y mutation affected the intrinsic nitrite reductase rate of T-state Hb independent of allostery. The three-fold increase in this rate in βF41Y is similar to the increase seen in fetal compared with adult Hb [41,42]; it is not clear if these differences have a kinetic or thermodynamic explanation.

The co-administration of nitrite has been suggested as being beneficial for maintaining NO levels following HBOC addition. However, enhanced metHb formation makes this problematic and *in vivo* results have not been promising [43]. The results from βF41Y suggest that — like NO dioxygenation — the nitrite reductase activity of Hb may be amenable to manipulation, raising the possibility of maintaining NO levels without enhancing metHb formation.

The idea of using βF41Y as part of a HBOC has been suggested previously [34]. However, the design rationale [35] was the engineering of a lower intrinsic oxygen affinity (by stabilizing the T-state), rather than enhancing the interaction with external reductants. The work here suggests that F41Y possesses an additional key property; in the presence of suitable reductants, βF41Y has superior antioxidative properties compared with wt Hb. This property is likely to be shared by other tyrosine mutations [17].

## Conclusion

In summary, the addition of a new surface-accessible tyrosine residue in the β-subunits renders Hb less oxidatively damaging without compromising its ability to act as an extracellular oxygen carrier. This enhanced reactivity with plasma antioxidants is not just a function of βF41Y in human Hb. We have shown even larger effects after introducing tyrosine residues to a variety of novel sites on the surface of *Aplysia* myoglobin [17]. Historically, the lack of a clinically viable HBOC product has been seen as due to adverse side effects rather than a lack of functionality in oxygen transport [44]. This report demonstrates that the introduction of new tyrosine electron transfer pathways in human Hb renders it less pro-oxidant. Alternative attempts to render Hb less oxidatively damaging have included cross-linking to antioxidant enzymes [45] or cellular antioxidants [46]. Protein engineering has the potential to deliver similar benefits in a more defined and homogenous product. We suggest that appropriate redox-active tyrosine mutations could form the basis for a new generation of HBOC that retain the functionality of previous products, but limit the adverse side effects.

## Abbreviations

Hb, hemoglobin; HBOC, hemoglobin-based oxygen carrier; HPODE, 13-S hydroperoxy 9-*cis*, 11-*trans* octadecadienoic acid; metHb, met(ferric) hemoglobin; NO, nitric oxide; oxyHb, oxygenated hemoglobin; SOSG, Singlet Oxygen Sensor Green; TFA, trifluoroacetic acid; TLR-4, Toll-like receptor 4; wt, wild-type recombinant.

## Author Contribution

C.E.C., M.T.W and B.J.R conceived and initiated the project. G.G.A.S performed the majority of experiments with help from R.S.S., M.S., K.K., K.R., L.R. and B.J.R. G.G.A.S. analysed the data with additional contributions from C.E.C., R.S.S., L.R., L.B., A.M. and B.J.R. C.E.C. and B.J.R. designed the experimental strategy. C.E.C. wrote the paper which was critically reviewed by G.G.A.S, B.J.R., A.M., L.R., L.B. and M.T.W.

## Funding

We thank the UK Biotechnology and Biological Sciences Research Council for financial support [BB/L004232/1].

## Competing Interests

C.E.C., B.J.R., and M.T.W. have a patent relating to modification of hemoglobin amino acids designed to render a blood substitute less toxic. C.E.C., B.J.R., M.T.W., and G.G.A.S. are shareholders in a related company (CymBlood).

## References

[1] Alayash A.I. (2014) Blood substitutes: why haven't we been more successful? Trends Biotechnol. 32, 177–185 doi:10.1016/j.tibtech.2014.02.006 24630491 PMC4418436

[2] Varnado C.L., Mollan T.L., Birukou I., Smith B.J.Z., Henderson D.P. and Olson J.S. (2013) Development of recombinant hemoglobin-based oxygen carriers. Antioxid. Redox Signal. 18, 2314–2328 doi:10.1089/ars.2012.4917 23025383 PMC3638513

[3] Dou Y., Maillett D.H., Eich R.F. and Olson J.S. (2002) Myoglobin as a model system for designing heme protein based blood substitutes. Biophys. Chem. 98, 127–148 doi:10.1016/S0301-4622(02)00090-X 12128195

[4] Looker D., Abbott-Brown D., Cozart P., Durfee S., Hoffman S., Mathews A.J. et al. (1992) A human recombinant haemoglobin designed for use as a blood substitute. Nature 356, 258–260 doi:10.1038/356258a0 1552945

[5] Komiyama N.H., Miyazaki G., Tame J. and Nagai K. (1995) Transplanting a unique allosteric effect from crocodile into human haemoglobin. Nature 373, 244–246 doi:10.1038/373244a0 7816138

[6] Baudin-Creuza V., Fablet C., Zal F., Green B.N., Promé D., Marden M.C. et al. (2002) Hemoglobin Porto Alegre forms a tetramer of tetramers superstructure. Protein Sci. 11, 129–136 doi:10.1110/ps.35702 11742129 PMC2368782

[7] Faggiano S., Bruno S., Ronda L., Pizzonia P., Pioselli B. and Mozzarelli A. (2011) Modulation of expression and polymerization of hemoglobin Polytaur, a potential blood substitute. Arch. Biochem. Biophys. 505, 42–47 doi:10.1016/j.abb.2010.09.027 20920461

[8] Levine J., Weickert M., Pagratis M., Etter J., Mathews A., Fattor T. et al. (1998) Identification of a nickel(II) binding site on hemoglobin which confers susceptibility to oxidative deamination and intramolecular cross-linking. J. Biol. Chem. 273, 13037–13046 doi:10.1074/jbc.273.21.13037 9582340

[9] Levine R.L., Mosoni L., Berlett B.S. and Stadtman E.R. (1996) Methionine residues as endogenous antioxidants in proteins. Proc. Natl Acad. Sci. USA 93, 15036–15040 doi:10.1073/pnas.93.26.15036 8986759 PMC26351

[10] Reeder B.J. (2010) The redox activity of hemoglobins: from physiologic functions to pathologic mechanisms. Antioxid. Redox Signal. 13, 1087–1123 doi:10.1089/ars.2009.2974 20170402

[11] Dunne J., Caron A., Menu P., Alayash A.I., Buehler P.W., Wilson M.T. et al. (2006) Ascorbate removes key precursors to oxidative damage by cell-free haemoglobin *in vitro* and *in vivo*. Biochem. J. 399, 513–524 doi:10.1042/BJ20060341 16848758 PMC1615907

[12] Cooper C.E., Jurd M., Nicholls P., Wankasi M.M., Svistunenko D.A., Reeder B.J. et al. (2005) On the formation, nature, stability and biological relevance of the primary reaction intermediates of myoglobins with hydrogen peroxide. Dalton Trans. 3483–3488 doi:10.1039/b505786h 16234929

[13] Matsunaga I. and Shiro Y. (2004) Peroxide-utilizing biocatalysts: structural and functional diversity of heme-containing enzymes. Curr. Opin. Chem. Biol. 8, 127–132 doi:10.1016/j.cbpa.2004.01.001 15062772

[14] Svistunenko D.A. (2005) Reaction of haem containing proteins and enzymes with hydroperoxides: the radical view. Biochim. Biophys. Acta, Bionerg. 1707, 127–155 doi:10.1016/j.bbabio.2005.01.004 15721611

[15] Reeder B.J., Cutruzzola F., Bigotti M.G., Hider R.C. and Wilson M.T. (2008) Tyrosine as a redox-active center in electron transfer to ferryl heme in globins. Free Radic. Biol. Med. 44, 274–283 doi:10.1016/j.freeradbiomed.2007.06.030 18215736

[16] Reeder B.J., Grey M., Silaghi-Dumitrescu R.-L., Svistunenko D.A., Bulow L., Cooper C.E. et al. (2008) Tyrosine residues as redox cofactors in human hemoglobin: implications for engineering nontoxic blood substitutes. J. Biol. Chem. 283, 30780–30787 doi:10.1074/jbc.M804709200 18728007 PMC2662160

[17] Reeder B.J., Svistunenko D.A., Cooper C.E. and Wilson M.T. (2012) Engineering tyrosine-based electron flow pathways in proteins: the case of *Aplysia* myoglobin. J. Am. Chem. Soc. 134, 7741–7749 doi:10.1021/ja211745g 22515641

[18] Burkert L.B., Sharma V.S., Pisciotta A.V., Ranney H.M. and Bruckheimer S. (1976) Hemoglobin M equon beta 41 (C7) phenylalanine leads to tyrosine. Blood 48, 645–651 PMID: 974262

[19] Antonini E. and Brunori M. (1971) Hemoglobin and Myoglobin and Their Reactions With Ligands, North Holland Publishing Company, Amsterdam

[20] Hayashi A., Suzuki T. and Shin M. (1973) An enzymic reduction system for metmyoglobin and methemoglobin, and its application to functional studies of oxygen carriers. Biochim. Biophys. Acta, Protein Struct. 310, 309–316 doi:10.1016/0005-2795(73)90110-4 4146292

[21] Ronda L., Bruno S., Faggiano S., Bettati S. and Mozzarelli A. (2008) Oxygen binding to heme proteins in solution, encapsulated in silica gels, and in the crystalline state. Methods Enzymol. 437, 311–328 doi:10.1016/S0076-6879(07)37016-X 18433635

[22] Reeder B.J. and Wilson M.T. (2005) Desferrioxamine inhibits production of cytotoxic heme to protein cross-linked myoglobin: a mechanism to protect against oxidative stress without iron chelation. Chem. Res. Toxicol. 18, 1004–1011 doi:10.1021/tx049660y 15962935

[23] Silkstone G., Kapetanaki S.M., Husu I., Vos M.H. and Wilson M.T. (2010) Nitric oxide binds to the proximal heme coordination site of the ferrocytochrome c/cardiolipin complex: formation mechanism and dynamics. J. Biol. Chem. 285, 19785–19792 doi:10.1074/jbc.M109.067736 20395293 PMC2888389

[24] Hargrove M.S., Singleton E.W., Quillin M.L., Ortiz L.A., Phillips G.N. Jr, Olson J.S. et al. (1994) His64(E7)-->Tyr apomyoglobin as a reagent for measuring rates of hemin dissociation. J. Biol. Chem. 269, 4207–4214 PMID: 8307983 10.2210/pdb1mgn/pdb

[25] Ascoli F., Fanelli M.R. and Antonini E. (1981) Preparation and properties of apohemoglobin and reconstituted hemoglobins. Methods Enzymol. 76, 72–87 doi:10.1016/0076-6879(81)76115-9 7329287

[26] Salhany J.M. (2008) Kinetics of reaction of nitrite with deoxy hemoglobin after rapid deoxygenation or predeoxygenation by dithionite measured in solution and bound to the cytoplasmic domain of band 3 (SLC4A1). Biochemistry 47, 6059–6072 doi:10.1021/bi8000819 18465875

[27] Vandegriff K.D., Malavalli A., Minn C., Jiang E., Lohman J., Young M.A. et al. (2006) Oxidation and haem loss kinetics of poly(ethylene glycol)-conjugated haemoglobin (MP4): dissociation between *in vitro* and *in vivo* oxidation rates. Biochem. J. 399, 463–471 doi:10.1042/BJ20060809 16813564 PMC1615902

[28] Doherty D.H., Doyle M.P., Curry S.R., Vali R.J., Fattor T.J., Olson J.S. et al. (1998) Rate of reaction with nitric oxide determines the hypertensive effect of cell-free hemoglobin. Nat. Biotechnol. 16, 672–676 doi:10.1038/nbt0798-672 9661203

[29] Reeder B.J. and Wilson M.T. (1998) Mechanism of reaction of myoglobin with the lipid hydroperoxide hydroperoxyoctadecadienoic acid. Biochem. J. 330, 1317–1323 doi:10.1042/bj3301317 9494102 PMC1219278

[30] Cooper C.E., Schaer D.J., Buehler P.W., Wilson M.T., Reeder B.J., Silkstone G. et al. (2013) Haptoglobin binding stabilizes hemoglobin ferryl iron and the globin radical on tyrosine β145. Antioxid. Redox Signal. 18, 2264–2273 doi:10.1089/ars.2012.4547.test 22702311 PMC3638561

[31] Ramirez D.C., Chen Y.-R. and Mason R.P. (2003) Immunochemical detection of hemoglobin-derived radicals formed by reaction with hydrogen peroxide: involvement of a protein-tyrosyl radical. Free Radic. Biol. Med. 34, 830–839 doi:10.1016/S0891-5849(02)01437-5 12654471

[32] Vallelian F., Garcia-Rubio I., Puglia M., Kahraman A., Deuel J.W., Engelsberger W.R. et al. (2015) Spin trapping combined with quantitative mass spectrometry defines free radical redistribution within the oxidized hemoglobin:haptoglobin complex. Free Radic. Biol. Med. 85, 259–268 doi:10.1016/j.freeradbiomed.2015.04.023 25933590

[33] Baudin-Creuza V., Vasseur-Godbillon C., Griffon N., Kister J., Kiger L., Poyart C. et al. (1999) Additive effects of β chain mutations in low oxygen affinity hemoglobin βF41Y,K66T. J. Biol. Chem. 274, 25550–25554 doi:10.1074/jbc.274.36.25550 10464287

[34] Baudin V., Pagnier J., Lacaze N., Bihoreau M.-T., Kister J., Marden M. et al. (1992) Allosteric properties of haemoglobin β41 (C7) Pheå Tyr: a stable, low-oxygen-affinity variant synthesized in *Escherichia coli*. Biochim. Biophys. Acta, Protein Struct. Mol. Enzymol. 1159, 223–226 doi:10.1016/0167-4838(92)90029-D 1390926

[35] Griffon N., Baudin V., Dieryck W., Dumoulin A., Pagnier J., Poyart C. et al. (1998) Tetramer-dimer equilibrium of oxyhemoglobin mutants determined from auto-oxidation rates. Protein Sci. 7, 673–680 doi:10.1002/pro.5560070316 9541399 PMC2143954

[36] Belcher J.D., Chen C., Nguyen J., Milbauer L., Abdulla F., Alayash A.I. et al. (2014) Heme triggers TLR4 signaling leading to endothelial cell activation and vaso-occlusion in murine sickle cell disease. Blood 123, 377–390 doi:10.1182/blood-2013-04-495887 24277079 PMC3894494

[37] Liong E.C., Dou Y., Scott E.E., Olson J.S. and Phillips G.N. Jr (2001) Waterproofing the heme pocket: role of proximal amino acid side chains in preventing hemin loss from myoglobin. J. Biol. Chem. 276, 9093–9100 doi:10.1074/jbc.M008593200 11084036

[38] Silkstone R.S., Silkstone G., Baath J.A., Rajagopal B., Nicholls P., Reeder B.J. et al. (2016) The βLys66Tyr variant of human hemoglobin as a component of a blood substitute. Adv. Exp. Med. Biol. 876, 455–460 doi:10.1007/978-1-4939-3023-4_57 26782245

[39] Grubina R., Huang Z., Shiva S., Joshi M.S., Azarov I., Basu S. et al. (2007) Concerted nitric oxide formation and release from the simultaneous reactions of nitrite with deoxy- and oxyhemoglobin. J. Biol. Chem. 282, 12916–12927 doi:10.1074/jbc.M700546200 17322300

[40] Rong Z., Wilson M.T. and Cooper C.E. (2013) A model for the nitric oxide producing nitrite reductase activity of hemoglobin as a function of oxygen saturation. Nitric Oxide 33, 74–80 doi:10.1016/j.niox.2013.06.008 23831540

[41] Blood A.B., Tiso M., Verma S.T., Lo J., Joshi M.S., Azarov I. et al. (2009) Increased nitrite reductase activity of fetal versus adult ovine hemoglobin. Am. J. Physiol. Heart Circ. Physiol. 296, H237–H246 doi:10.1152/ajpheart.00601.2008 19028797 PMC2643889

[42] Rong Z., Alayash A.I., Wilson M.T. and Cooper C.E. (2013) Modulating hemoglobin nitrite reductase activity through allostery: a mathematical model. Nitric Oxide 35, 193–198 doi:10.1016/j.niox.2013.10.007 24177061

[43] Moon-Massat P., Scultetus A., Arnaud F., Brown A., Haque A., Saha B. et al. (2012) The effect HBOC-201 and sodium nitrite resuscitation after uncontrolled haemorrhagic shock in swine. Injury 43, 638–647 doi:10.1016/j.injury.2010.10.013 21094491

[44] Silverman T.A. and Weiskopf R.B. (2009) Hemoglobin-based oxygen carriers: current status and future directions. Transfusion 49, 2495–2515 doi:10.1111/j.1537-2995.2009.02356.x 19903298

[45] D'Agnillo F. and Chang T.M. (1998) Polyhemoglobin-superoxide dismutase-catalase as a blood substitute with antioxidant properties. Nat. Biotechnol. 16, 667–671 doi:10.1038/nbt0798-667 9661202

[46] Simoni J., Simoni G., Wesson D.E., Griswold J.A. and Feola M. (2000) A novel hemoglobin-adenosine-glutathione based blood substitute: evaluation of its effects on human blood ex vivo. ASAIO J. 46, 679–692 doi:10.1097/00002480-200011000-00007 11110264

